# Commentary: FUS affects circular RNA expression in murine embryonic stem cell-derived motor neurons

**DOI:** 10.3389/fnmol.2017.00412

**Published:** 2017-12-12

**Authors:** Bert M. Verheijen, R. Jeroen Pasterkamp

**Affiliations:** Department of Translational Neuroscience, Brain Center Rudolf Magnus, University Medical Center Utrecht, Utrecht University, Utrecht, Netherlands

**Keywords:** circular RNA, RNA-binding protein, splicing, FUS, motor neuron disease

Circular RNAs (circRNAs) are a class of single-stranded RNA characterized by a covalently closed loop structure, lacking 5′-3′ polarity. They are generated through “back-splicing” events, wherein a downstream 5′ splice site is joined to an upstream 3′ splice site (Wu et al., 2017) (Figure 1A). Their remarkable structure provides circRNAs with properties that distinguish them from their linear counterparts. For example, due to absence of 5′ and 3′ ends circRNAs are resistant to exonuclease-mediated degradation and are presumably more stable than many linear RNAs.

**Figure 1:**
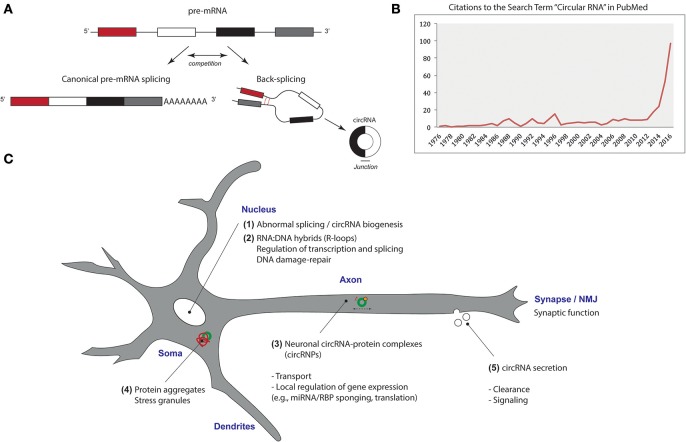
Circular RNAs and motor neuron disease. **(A)** Circular RNAs (circRNAs) are generated in a “non-canonical” splicing event (Sibley et al., 2016), referred to as “back-splicing.” In back-splicing reactions, a downstream 5′ splice site (splice donor) is joined to an upstream 3′ splice site (splice acceptor) to form a covalently closed loop structure. A horizontal line indicates the head-to-tail junction of the circRNA. circRNAs have biochemical properties that distinguish them from linear RNA species. For example, they are highly resistant to exonucleases, because they lack free 5′ and 3′ ends. Treatment of RNA with the magnesium-dependent 3′->5′exoribonuclease RNAse R efficiently digests linear RNA, but not circRNA (Suzuki et al., 2006; Chu et al., 2017). **(B)** In recent years, it has become clear that circRNAs are widely expressed in cells and that some may have specific biological functions. This has resulted in profound interest in circRNAs as supported by an increasing number of publications listed in PubMed. **(C)** circRNAs have been shown to be deregulated in motor neuron disease **(1)** and might carry out specific roles in motor neurons. Potential functions of circRNAs include the regulation of transcription and pre-mRNA splicing **(2)**. A study in *Arabidopsis* has shown that a circRNA can interact with its cognate DNA locus via complementary base-pairing to produce a DNA-RNA hybrid, or R-loop, to promote alternative splicing of its corresponding mRNA, altering floral morphology (Conn et al., 2017). Aberrant circRNA-DNA interactions may be partly responsible for neuronal abnormalities, e.g., elevated levels of R-loops have been found in motor neuron disease models (e.g., Walker et al., 2017). Additionally, circRNAs are involved in circRNA-protein (circRNP) complex formation and localization, sponging of other RNAs (e.g., miRNAs) and RNA-binding proteins (RBPs), and may be translated into proteins/peptides **(3)**. circRNAs may control oscillations in transcriptional networks (e.g., as molecular components of the circadian clock) and influence processes like pluripotency maintenance and neuronal differentiation (You et al., 2015; Yu et al., 2017). In addition, circRNAs might play roles in the formation of aggregates and stress granules **(4)** and can also be secreted, perhaps acting as trans-acting signaling molecules **(5)**.

Although the existence of circRNAs was first reported several decades ago, these RNAs were long considered to be a curiosity (Ares, 2015). However, recent studies have revealed circRNAs to be ubiquitous components of eukaryotic gene expression with gene regulatory functions (Salzman et al., 2012; Jeck et al., 2013; Wang et al., 2014; Szabo and Salzman, 2016). For example, some circRNAs bind and sponge microRNAs (miRNAs), thereby regulating their actions (Hansen et al., 2013; Memczak et al., 2013). In addition, some circRNAs may be translated (Legnini et al., 2017; Pamudurti et al., 2017). This recent progress has caused a surge of interest into circRNAs (Figure 1B). However, the biogenesis and the physiological functions of most circRNAs remain largely unknown.

circRNAs are particularly abundant in the nervous system, showing highly specific, conserved, and dynamic expression patterns in neurons (Rybak-Wolf et al., 2015; Venø et al., 2015; You et al., 2015; Zappulo et al., 2017). This hints at important roles for these RNAs in neuronal tissue (Chen and Schuman, 2016; van Rossum et al., 2016). It was recently reported that removal of the *Cdr1as* locus in mice, which encodes the extraordinary “miRNA super-sponge” circRNA Cdr1as (Barrett et al., 2017), results in deregulation of specific miRNAs and affects brain function (Piwecka et al., 2017). In addition, circRNAs have been associated with neural disorders, such as Alzheimer's disease (Lukiw, 2013).

Now, Errichelli et al. describe the effects of the RNA-binding protein (RBP) fused in sarcoma (FUS) on circRNA expression (Errichelli et al., 2017). RNA splicing is highly context-dependent and multiple RBPs regulate this process (Fu and Ares, 2014). Not surprisingly, RBPs can influence circRNA biogenesis, as has been previously shown for the splicing factors Muscleblind and Quaking, and for several hnRNPs and SR proteins (Ashwal-Fluss et al., 2014; Conn et al., 2015; Kramer et al., 2015). FUS regulates several RNA metabolic processes, including RNA splicing, and is linked to the pathogenesis of amyotrophic lateral sclerosis (ALS) and frontotemporal dementia (FTD) (Robberecht and Philips, 2013; Scotti and Swanson, 2016; van Es et al., 2017). Therefore, insight into the roles of FUS on circRNA expression could provide new clues on how circRNAs are formed and how circRNAs contribute to FUS-associated neurological disorders.

In their study, Errichelli et al. identified circRNAs in *in vitro*-derived mouse motor neurons (purified mouse embryonic stem cell (mESC)-derived motor neurons, obtained from wild-type (*FUS*^+/+^) or knockout (*FUS*^−/−^) mice) and demonstrate that FUS, through its control of back-splicing reactions, regulates the production of a considerable number of circRNAs.

Similar to altered circRNA expression in FUS^−/−^-mESC-derived motor neurons, as determined by RNA-Seq (their Figures 1, 2, their Tables 1, 2), FUS depletion (siFUS) in a neuroblastoma cell line consistently affected circRNA expression. Aberrant circRNA expression was rescued upon ectopic expression of wild-type, but not ALS-associated mutant FUS (FUS^R521C^, FUS^P525L^) [specifically not of those that were downregulated after FUS knockdown (their Figure 3)]. Interestingly, human-induced pluripotent stem cell (hiPSC)-derived motor neurons harboring ALS-linked FUS mutations (FUS^P525L^) also showed deregulation of specific circRNAs, some of which are conserved between mouse and human. These findings suggest that mutation of FUS affects circRNA biogenesis. In future studies, it will be interesting to see how changes in the subcellular localization of FUS or in binding of interaction partners (RNA, protein) as seen in neurological disease can influence circRNAs.

The FUS-dependent effect on circRNA generation appears, at least in part, to be regulated by binding of FUS to introns flanking back-splice junctions. Cross-linking immunoprecipitation (CLIP) analysis demonstrated enriched FUS binding on circularizing exon–intron regions (their Figure 4). Additionally, a direct effect of FUS on circRNA biogenesis was shown in elegant experiments using artificial circRNA expression constructs, containing two regions spanning intron 1 (~1,500 nt)–exon 2 and exon 3–intron 2 (~1,500 nt) of select host genes (their Figure 5).

This study by Errichelli et al. is an exciting step forward in dissecting the contribution of specific RBPs to circRNA biogenesis and in exploring deregulation of circRNAs in motor neuron disease. It is anticipated that upcoming studies will shed light on the potential roles of other disease-related RBPs (e.g., TDP-43, SMN, ATXN2) in circRNA formation. Errichelli et al. provide an excellent experimental framework for this purpose. The presented findings also raise several new questions.

For example, does the deregulation circRNA contribute to functional defects causing motor neuron disease? Whether and how many neuronal circRNAs have specific functions remains largely unknown, but recent work indicates roles for circRNAs in various cellular processes, including those affected in motor neuron disease (Figure 1C). Such deficits include (local) deregulation of other RNAs, e.g., miRNAs (Rotem et al., 2017). Furthermore, circRNAs are present in circRNA-protein complexes (circRNPs) (Schneider et al., 2016), which are likely to be important for neuronal transport, localization of RNA and proteins, and control of local protein synthesis, e.g., in growth cones or at neuromuscular synapses. RBPs, like FUS, therefore most likely affect circRNA function beyond their biogenesis, e.g., through interactions in the cytoplasm and by recruiting circRNAs to protein aggregates or stress granules (Blokhuis et al., 2013). For example, although Errichelli et al. present evidence that FUS may directly bind intronic sequences to affect circRNA biogenesis, it will also be important to determine potential indirect effects of FUS mutation, e.g., by possible changes in general RNA splicing and expression of other RNAs (e.g., miRNAs). Future studies should explore the link between RBP levels and mutations, pre-mRNA splicing defects, circRNAs, and compartment-specific functional impairments in more detail in different experimental models.

New insights into context-specific circRNA biogenesis via RBPs will probably not only contribute to our understanding of circRNAs in neuronal disorders and lead to the identification of novel molecular biomarkers and therapeutic targets, but may also provide clues about their physiological functions, e.g., in RNA-based mechanisms underlying neuronal development.

## Author contributions

BV drafted Figure 1. BV and RP wrote the manuscript.

### Conflict of interest statement

The authors declare that the research was conducted in the absence of any commercial or financial relationships that could be construed as a potential conflict of interest.
